# Digital Sleep Disruption: Unraveling the Network Structure of Technology Use and Sleep Problems Through Network Analysis

**DOI:** 10.1002/cns.70778

**Published:** 2026-02-16

**Authors:** Haoqing Gan, Lingjia Xu, Chenglin Tong

**Affiliations:** ^1^ Department of Emergency Shaoxing Second Hospital Shaoxing Zhejiang China; ^2^ School of Medicine Shaoxing University Shaoxing Zhejiang China; ^3^ Department of Neurology Shaoxing Second Hospital Shaoxing Zhejiang China

**Keywords:** blue light exposure, circadian rhythm, digital technology, network analysis, psychological mechanisms, sleep problems

## Abstract

**Background:**

Sleep problems have emerged as a critical health concern in the digital age, yet the complex mechanisms linking technology use to sleep disruption remain poorly understood. Previous research has typically examined isolated relationships between specific technological behaviors and sleep outcomes, overlooking the complex interplay among various digital factors. This study aims to address this gap by employing network analysis to investigate the interconnected relationships among multiple technology‐related factors and their collective influence on sleep problems.

**Methods:**

Using network analysis, this study examined how different aspects of digital technology use collectively influence sleep problems in a large sample of Chinese adults (*N* = 9443). Participants were recruited through stratified random sampling based on age groups and geographical regions, with the sample size determined a priori using Monte Carlo simulations to ensure stable network estimation. Participants completed validated measures assessing screen time, before‐bed electronic device use, electronic device dependency, social media anxiety, digital information overload, virtual social pressure, blue light exposure, circadian rhythm disruption, online gaming addiction, work‐life digital integration, and sleep problems. Participants with diagnosed sleep disorders, those engaged in shift work, or those who had traveled across time zones in the past month were excluded to minimize confounding effects on natural sleep patterns.

**Results:**

Network analysis revealed complex interconnections among technology‐related factors and sleep problems. Blue light exposure demonstrated the strongest direct edge weight with sleep problems (*r* = 0.31, *p* < 0.001), followed by circadian rhythm disturbance (*r* = 0.26, *p* < 0.001). The network structure indicated that screen time, bedtime device use, electronic device dependence, virtual social pressure, and work‐life digital integration showed weaker direct associations with sleep problems but demonstrated substantial indirect pathways through intermediate variables. Online gaming addiction, digital information overload, social media anxiety, and circadian rhythm disturbance exhibited moderate centrality indices, suggesting their role as potential mediators within the network.

**Conclusions:**

These findings advance our understanding of technology‐induced sleep disruption and suggest potential targets for intervention. The network approach reveals that addressing sleep problems in the digital age requires consideration of both direct physiological impacts and indirect psychological pathways. The identification of specific factors with high centrality provides empirical guidance for developing targeted intervention strategies.

## Graphical abstract

In 9443 Chinese adults, network analysis reveals blue light exposure as the central factor most strongly associated with sleep problems (*r* = 0.31). General screen time shows minimal direct effects (*r* = 0.03), with sleep disruption mediated through evening‐specific behaviors. These findings suggest interventions targeting blue light exposure and circadian rhythms may be more effective than general screen time reduction.

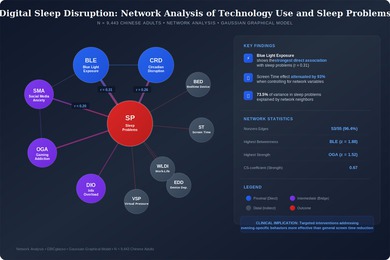

**Abbreviations**
BED: Before‐bed Electronic Device Use Scale
BLE: Blue Light Exposure Scale
CRD: Circadian Rhythm Disruption Scale
CS: Correlation Stability
DIO: Digital Information Overload Scale
EBIC: Extended Bayesian Information Criterion
EDD: Electronic Device Dependency Scale
GGM: Gaussian Graphical Model
ISI: Insomnia Severity Index
OGA: Online Gaming Addiction Scale
PSQI: Pittsburgh Sleep Quality Index
SMA: Social Media Anxiety Scale
SP: Sleep Problems
ST: Screen Time Scale
VSP: Virtual Social Pressure Scale
WLDI: Work‐Life Digital Integration Scale

## 1 Background

The pervasive integration of digital technology into daily life has fundamentally altered human sleep patterns, presenting an unprecedented challenge to public health. Recent epidemiological data indicate that sleep problems now affect over 45% of adults in technologically advanced societies, with prevalence rates showing a concerning upward trend [1]. This increase parallels the dramatic rise in digital technology use, with average daily screen time exceeding 8 h in many populations, which has been associated with increased sleep disturbances and circadian disruption [2]. The convergence of these trends raises critical questions about how different aspects of digital technology use collectively influence sleep health in the modern era.

The relationship between technology use and sleep problems has been extensively studied from various perspectives, drawing on theoretical frameworks from chronobiology, cognitive psychology, and behavioral medicine. Chronobiological research has demonstrated that evening exposure to blue light from digital devices can suppress melatonin production and delay sleep onset by up to 3 h [2, 3]. This effect is mediated through intrinsically photosensitive retinal ganglion cells (ipRGCs) that project to the suprachiasmatic nucleus, the master circadian pacemaker [4]. Psychological studies have revealed that social media use and digital communication can create states of cognitive and emotional arousal that persist well beyond actual device use, interfering with the psychological wind‐down process necessary for sleep initiation [5, 6]. Behavioral research has documented how technology use can disrupt sleep through delayed bedtimes, irregular sleep schedules, and suboptimal pre‐sleep routines including device use in bed, bright screen exposure, and engagement with stimulating content [7].

Despite this extensive research, our understanding of technology‐induced sleep disruption remains fragmented and incomplete. Current models often fail to capture the complex, interconnected nature of modern digital behavior and its impact on sleep. For instance, while the direct effects of blue light exposure on circadian rhythms are well‐established through physiological mechanisms, this pathway likely interacts with psychological factors such as social media anxiety and digital information overload through both shared variance and unique contributions that current unifactorial models cannot adequately explain [8].

Several critical gaps in the literature warrant attention. First, most studies examine isolated relationships between specific technological behaviors and sleep outcomes, overlooking potential interaction effects and indirect pathways that cannot be captured by simple bivariate models or traditional regression approaches [9].

Second, the role of psychological mechanisms in mediating technology‐sleep relationships remains poorly understood. While research has identified associations between digital technology use and various psychological states—such as social media anxiety, information overload, and virtual social pressure—how these psychological factors collectively influence sleep problems through interconnected pathways requires deeper investigation [10]. Recent theoretical work suggests that psychological arousal may play a crucial role in translating digital behavior into sleep disruption through multiple mechanisms, including pre‐sleep cognitive hyperarousal, emotional dysregulation, and disrupted relaxation processes [11].

Third, existing research often treats technology use as a static construct, failing to account for the dynamic and multifaceted nature of modern digital behavior. Ecological momentary assessment studies indicate that different patterns of technology use may have distinct impacts on sleep, with factors such as timing, content type, and social context all potentially moderating the relationship [12]. Understanding these nuanced relationships requires more sophisticated analytical approaches that can capture the complex interplay between multiple technological factors while accounting for their shared and unique contributions.

Network analysis offers a promising framework for addressing these limitations. Rooted in graph theory and complex systems science, this approach conceptualizes psychological phenomena as systems of interacting components, allowing examination of how multiple factors simultaneously influence each other while controlling for all other variables in the network [13]. Unlike traditional regression approaches that assume predictors are independent, network analysis explicitly models the interdependencies among variables, providing a more ecologically valid representation of how multiple factors coexist and interact. In the context of technology‐induced sleep disruption, network analysis can identify central symptoms that might serve as optimal intervention targets, map indirect pathways through which technological factors affect sleep, detect potential feedback loops between variables, and quantify the overall connectivity of the system [14].

Recent applications of network analysis in psychological and health research have yielded important insights into the structure of various phenomena. Studies examining anxiety disorders, depression, and behavioral addictions have demonstrated the utility of network approaches in understanding complex psychological systems and identifying intervention targets [15, 16]. For example, research by Abaas et al. [6] demonstrated the interconnected nature of psychological symptoms related to social media use, showing that female participants exhibited higher levels of anxiety and depression, illustrating how network‐based approaches can reveal the complex relationships among technology use, psychological symptoms, and health outcomes. However, network analysis remains underutilized in understanding technology‐induced sleep disruption, despite the inherently interconnected nature of digital behavior and sleep problems.

The present study employs network analysis to examine how different aspects of digital technology use collectively influence sleep problems in a large sample of Chinese adults. Drawing on recent theoretical and empirical work, we conceptualize the relationship between digital technology use and sleep problems as a network system comprising proximal factors that directly impact sleep physiology, intermediate factors that bridge distal behaviors and sleep outcomes, and distal factors that represent broader patterns of digital engagement. This differentiation allows us to investigate not only which factors most strongly associate with sleep problems but also the structural properties of the technology‐sleep network and the pathways through which effects may operate.

Based on theoretical frameworks from chronobiology, cognitive science, and digital behavior research, we propose three hypotheses:

*Blue light exposure will demonstrate the strongest direct edge weight with sleep problems in the network, reflecting its established physiological impact on circadian regulation and melatonin suppression*.

*Screen time, bedtime device use, electronic device dependence, virtual social pressure, and work‐life digital integration will demonstrate weaker direct associations with sleep problems but stronger indirect pathways through intermediate variables, reflecting their role as distal factors*.

*Online gaming addiction, digital information overload, social media anxiety, and circadian rhythm disturbance will demonstrate moderate centrality indices, indicating their potential role as bridge symptoms connecting distal factors to sleep outcomes*.

## 2 Methods

### 2.1 Participants and Sampling Procedure

Data were collected from 9443 Chinese adults (54.3% female) aged 18–65 years (*M* = 28.4, SD = 8.7) through the Credamo platform between September 2023 and January 2024. Participants were recruited using stratified random sampling to ensure demographic representativeness. The stratification variables included age group (18–25, 26–35, 36–45, 46–55, 56–65 years) and geographical region (Eastern, Central, Western, and Northeastern China), with quotas set to approximate the population distribution according to the most recent national census data. Within each stratum, participants were randomly selected from the Credamo panel, which comprises over 2.6 million registered users across China.

The sample size was determined a priori based on recommendations for stable network estimation in psychological research. Following guidelines established by Epskamp et al. [17], we employed Monte Carlo simulations to estimate the minimum sample size required to detect edges with adequate power (≥ 0.80) and precision (correlation stability coefficient ≥ 0.25). Based on these simulations, which assumed a sparse network with approximately 50–60 edges and medium effect sizes, a minimum sample of 500 participants was required. However, to ensure robust estimation of centrality indices and to enable subgroup analyses, we targeted a substantially larger sample. The final sample of 9443 participants provides excellent statistical power and stable network estimates.

Inclusion criteria required regular use of digital devices (> 2 h daily) and proficiency in reading Chinese. Exclusion criteria included: (1) diagnosed sleep disorders (e.g., obstructive sleep apnea, restless leg syndrome, narcolepsy) in the past month, as such conditions represent distinct clinical entities with established pathophysiology that may confound the examination of technology‐related sleep disruption; (2) current shift work or rotating work schedules, which independently disrupt circadian rhythms through mechanisms unrelated to technology use; (3) trans‐meridian travel crossing three or more time zones in the past month, which can induce jet lag and temporary circadian misalignment; and (4) current use of medications known to affect sleep architecture (e.g., sedative‐hypnotics, stimulants, certain antidepressants). These exclusion criteria were implemented to isolate the effects of technology‐related factors on sleep problems while minimizing confounding from established medical conditions and environmental factors with independent effects on sleep.

### 2.2 Measures

All measures underwent rigorous translation and back‐translation following established guidelines [18]. For scales originally developed in English, the forward translation was conducted by two bilingual researchers independently, followed by reconciliation and back‐translation by a third researcher blind to the original version. Discrepancies were resolved through discussion with content experts. Responses used 5‐point Likert scales (1 = strongly disagree to 5 = strongly agree) unless otherwise specified.

#### 2.2.1 The Screen Time Scale (ST)

This 8‐item scale assesses daily duration and patterns of digital device use across various contexts (work, leisure, social). The scale was adapted from validated measures of technology use [19] and demonstrated good internal consistency (McDonald's *ω* = 0.89) and 2‐week test–retest reliability (*r* = 0.85). Sample items included “I spend more time using digital devices than intended” and “My screen time interferes with daily activities.”

#### 2.2.2 The Before‐Bed Electronic Device Use Scale (BED)

This 6‐item scale measures technology use patterns specifically during the 2‐h window before sleep (*ω* = 0.85). Items assessed behaviors such as device use while in bed, immediate pre‐sleep technology engagement, and the types of activities performed. The scale showed strong convergent validity with 7‐day diary measures of evening technology use (*r* = 0.78).

#### 2.2.3 The Electronic Device Dependency Scale (EDD)

This 9‐item scale evaluates psychological dependence on digital devices (*ω* = 0.91). Based on behavioral addiction criteria from the DSM‐5, the scale assessed symptoms such as loss of control over use, withdrawal symptoms when unable to use devices, tolerance, and continued use despite negative consequences. The scale has been validated against clinical assessments of problematic technology use [20].

#### 2.2.4 The Social Media Anxiety Scale (SMA)

This 10‐item scale measures anxiety specifically related to social media use (*ω* = 0.92). Items assessed concerns about missing updates (fear of missing out), compulsive checking behavior, social comparison anxiety, and anxiety about online self‐presentation. The scale demonstrated good discriminant validity from general anxiety measures (*r* = 0.45 with the GAD‐7), indicating that it captures a distinct construct.

#### 2.2.5 The Digital Information Overload Scale (DIO)

This 8‐item scale assesses perceived overwhelm from digital information sources (*ω* = 0.88). Items measured difficulty managing the flow of digital information, cognitive overload symptoms, and associated stress responses. The scale was adapted from established measures of information overload [21] and has been validated in Chinese populations [22].

#### 2.2.6 The Virtual Social Pressure Scale (VSP)

This 8‐item scale evaluates perceived pressure from digital social interactions (*ω* = 0.86). Items assessed feelings of obligation to maintain an online presence, pressure to respond quickly to digital communications, and anxiety about social expectations in virtual environments.

#### 2.2.7 The Blue Light Exposure Scale (BLE)

This 5‐item scale measures exposure to device‐emitted blue light (*ω* = 0.83), focusing on evening exposure patterns, device brightness settings, and use of blue light filtering features. The scale demonstrated significant correlations (*r* = 0.62) with objective measures of evening light exposure obtained through light sensors worn by a validation subsample (*n* = 120), supporting its criterion validity [23].

#### 2.2.8 The Circadian Rhythm Disruption Scale (CRD)

This 7‐item scale assesses disruption of sleep–wake patterns (*ω* = 0.87). Items evaluated irregularity in sleep timing, social jetlag (difference between weekday and weekend sleep schedules), and misalignment between preferred and actual sleep schedules. The scale was developed based on established circadian measures [24] and correlated significantly with actigraphy‐derived measures of sleep regularity in validation studies.

#### 2.2.9 The Online Gaming Addiction Scale (OGA)

This 10‐item scale measures problematic gaming behavior (*ω* = 0.93), including items assessing loss of time control, gaming interference with sleep and daily activities, and continued gaming despite negative consequences. The scale was based on the Internet Gaming Disorder criteria [25] and has been validated in Chinese populations.

#### 2.2.10 The Work‐Life Digital Integration Scale (WLDI)

This 7‐item scale evaluates the extent of work‐related technology use outside normal working hours (*ω* = 0.84), particularly focusing on evening work‐related digital behavior, expectations of availability, and boundary management between work and personal digital use.

#### 2.2.11 The Sleep Problems Scale (SP)

This 12‐item scale assesses various aspects of sleep disruption (*ω* = 0.94). Based on the Pittsburgh Sleep Quality Index (PSQI) [26] and the Insomnia Severity Index (ISI) [27], the scale evaluated difficulties with sleep initiation, sleep maintenance, early morning awakening, and perceived sleep quality. The scale demonstrated strong correlations with PSQI global scores (*r* = 0.82) and with objective sleep measures (sleep efficiency, *r* = −0.58; wake after sleep onset, *r* = 0.54) obtained through actigraphy in a validation subsample.

### 2.3 Procedure and Data Quality

Participants completed the online survey through the Credamo platform. The survey took approximately 20–25 min to complete. Data quality was ensured through multiple mechanisms: (1) three attention check items distributed throughout the survey (e.g., “Please select ‘Agree’ for this item”); (2) response time monitoring with exclusion of responses completed in less than 8 min (indicating inattentive responding); (3) consistency checks for logically related items; and (4) exclusion of responses with excessive missing data (> 10% of items). These quality control procedures resulted in the exclusion of 847 responses (8.2% of initial completions), yielding the final sample of 9443 participants.

### 2.4 Statistical Analysis

Network analysis was conducted using R software (version 4.2.3) with the qgraph [28], bootnet [17], and mgm [29] packages. A regularized partial correlation network was estimated using the graphical LASSO (least absolute shrinkage and selection operator) method combined with the Extended Bayesian Information Criterion (EBIC) for model selection. This approach produces a sparse network where edges represent unique associations between variables after controlling for all other variables in the network—a critical advantage over simple correlation matrices that conflate direct and indirect relationships.

In this model, known as the Gaussian Graphical Model (GGM), nodes represent observed variables (composite scores from each scale), while edges denote partial correlations between two nodes after accounting for all other nodes in the network. This statistical property is crucial for interpretation: edge weights reflect the unique association between two variables that cannot be explained by any other variable in the network. Positive associations are indicated by green edges, and negative associations by red edges, with edge thickness reflecting the strength of partial correlations.

To assess node importance, we calculated three centrality indices using the centralityPlot function from qgraph [28]: (1) Strength centrality, defined as the sum of absolute edge weights connected to a node, indicating the node's overall connectivity; (2) Betweenness centrality, reflecting the number of times a node lies on the shortest path between two other nodes, indicating its potential as a bridge; and (3) Closeness centrality, calculated as the inverse of the average shortest path length from a node to all other nodes, indicating how quickly changes in one node might spread to others.

Given the relative nature of centrality metrics, we additionally employed the mgm package to estimate node predictability (*R* ^2^), which quantifies the proportion of variance in each node explained by its neighboring nodes [29]. Unlike centrality indices, predictability provides an absolute measure of interconnectedness that is comparable across studies and indicates how much of each node's variance is accounted for by the network structure.

Network stability and accuracy were evaluated using a case‐dropping bootstrap procedure with 1000 iterations [17]. This procedure estimates the correlation stability (CS) coefficient, which indicates the maximum proportion of cases that can be dropped while maintaining a correlation of 0.7 between the original centrality indices and those from the bootstrap samples. A CS coefficient above 0.25 is considered acceptable, and above 0.5 is considered good. Edge weight accuracy was assessed through 95% confidence intervals derived from nonparametric bootstrapping.

## 3 Results

### 3.1 Network Structure and Edge Weights

**FIGURE 1:**
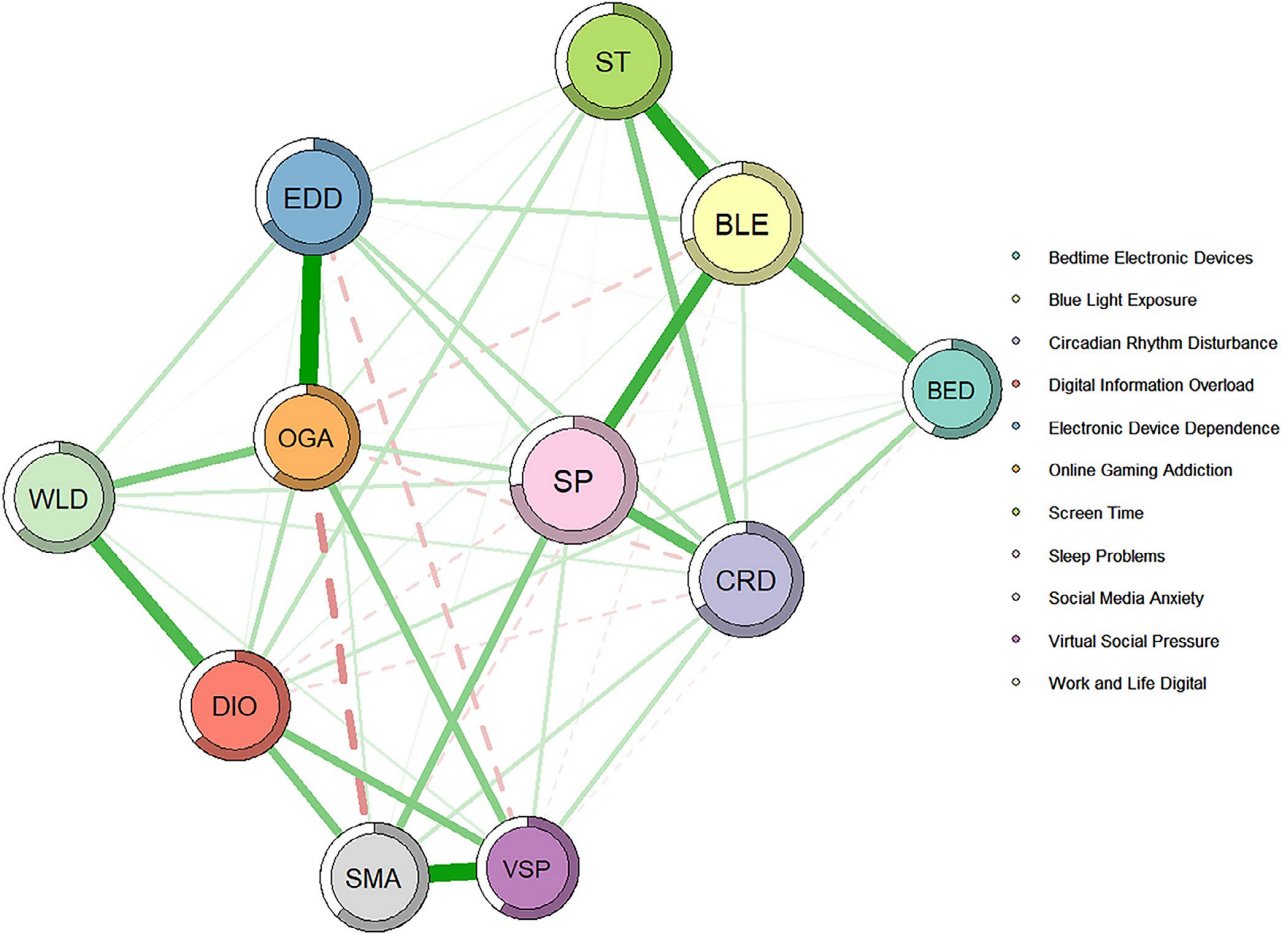
Sleep problems network EBICglasso model with predictability indicators.

Figure 1 presents the EBICglasso network model estimation for Sleep Problems (SP), comprising 11 nodes. Across the entire network, 53 out of 55 possible edges (96.4%) were nonzero, indicating a highly connected network structure. The strongest positive edge weight in the network was observed between Electronic Device Dependence (EDD) and Online Gaming Addiction (OGA) (*r* = 0.41, *p* < 0.001), while the strongest negative edge weight was between Social Media Anxiety (SMA) and Online Gaming Addiction (OGA) (*r* = −0.19, *p* < 0.001).

The network model comprises 11 nodes representing technology use factors and sleep problems. Each node is labeled with its abbreviation: SP = Sleep Problems; ST = Screen Time; BED = Before‐bed Electronic Device Use; EDD = Electronic Device Dependency; SMA = Social Media Anxiety; DIO = Digital Information Overload; VSP = Virtual Social Pressure; BLE = Blue Light Exposure; CRD = Circadian Rhythm Disruption; OGA = Online Gaming Addiction; WLDI = Work‐Life Digital Integration. Green edges indicate positive partial correlations, while red edges indicate negative partial correlations. Edge thickness reflects the magnitude of partial correlations (edge weights). The colored ring surrounding each node represents node predictability (*R* ^2^), indicating the proportion of variance explained by neighboring nodes.

Among all nonzero edges connected to the SP node, the strongest positive edge weights were with Blue Light Exposure (BLE) (*r* = 0.31, *p* < 0.001) and Circadian Rhythm Disturbance (CRD) (*r* = 0.26, *p* < 0.001), followed by Social Media Anxiety (SMA) (*r* = 0.20, *p* < 0.001), Electronic Device Dependence (EDD) (*r* = 0.12, *p* < 0.001), and Online Gaming Addiction (OGA) (*r* = 0.12, *p* < 0.001). Weaker positive edge weights were found between SP and Virtual Social Pressure (VSP) (*r* = 0.09, *p* < 0.001), Work‐Life Digital Integration (WLDI) (*r* = 0.09, *p* < 0.001), Bedtime Electronic Device Use (BED) (*r* = 0.05, *p* = 0.002), and Screen Time (ST) (*r* = 0.03, *p* = 0.041). Digital Information Overload (DIO) showed a small negative edge weight with SP (*r* = −0.07, *p* < 0.001).

### 3.2 Comparison of Correlation and Partial Correlation Matrices

A systematic comparison between the zero‐order correlation matrix and the partial correlation (edge weight) matrix revealed substantial attenuation of associations when controlling for other network variables (see Table 1). For example, the zero‐order correlation between Screen Time (ST) and Sleep Problems (SP) was *r* = 0.42, whereas the corresponding edge weight in the network was *r* = 0.03—a reduction of 93%. Similarly, Bedtime Electronic Device Use (BED) showed a zero‐order correlation of *r* = 0.38 with SP but an edge weight of only *r* = 0.05 (87% reduction). In contrast, Blue Light Exposure (BLE) showed a zero‐order correlation of *r* = 0.52 with SP and maintained a substantial edge weight of *r* = 0.31 (40% reduction). This pattern indicates that much of the variance shared between distal technology factors (ST, BED) and sleep problems is explained by other variables in the network, particularly intermediate factors like CRD and BLE. The relatively preserved edge weight for BLE suggests that its association with SP reflects a more direct relationship that is not fully accounted for by other network variables.

**TABLE 1:** Node predictability and centrality indices for the sleep problems network.

Variable	Predictability (*R* ^2^)	Betweenness	Closeness	Strength
Screen Time (ST)	0.67	−0.67	−0.66	−0.96
Bedtime Electronic Device Use (BED)	0.57	−0.95	−1.58	−2.00
Social Media Anxiety (SMA)	0.63	0.46	1.33	0.69
Digital Information Overload (DIO)	0.63	0.18	−0.15	0.15
Circadian Rhythm Disturbance (CRD)	0.66	−0.95	−0.27	0.05
Electronic Device Dependence (EDD)	0.67	−0.10	0.11	−0.08
Blue Light Exposure (BLE)	0.70	1.88	0.31	0.91
Virtual Social Pressure (VSP)	0.59	−0.67	0.08	0.34
Work‐Life Digital Integration (WLDI)	0.64	−0.95	−1.26	−1.06
Online Gaming Addiction (OGA)	0.61	0.18	0.22	1.52
Sleep Problems (SP)	0.74	1.60	1.86	0.46

*Note:* Predictability (*R* ^2^) represents the proportion of variance in each node explained by all its neighboring nodes in the network. Centrality measures (Betweenness, Closeness, and Strength) are presented as standardized z‐scores, with higher values indicating greater centrality.

### 3.3 Regarding Blue Light Exposure and Circadian Rhythm Disturbance

The edge weight between BLE and CRD was moderate (*r* = 0.18, *p* < 0.001), which may appear counterintuitive given the established physiological pathway through which blue light affects circadian rhythms. These findings warrant explanation. First, the network model estimates partial correlations controlling for all other variables, and the shared variance between BLE and CRD is partly explained by their mutual associations with other network nodes (particularly BED and EDD). Second, the BLE scale primarily captured behavioral patterns of light exposure (timing, brightness settings), while the CRD scale assessed subjective experiences of circadian misalignment; these represent different levels of the causal pathway. Third, individual differences in circadian photosensitivity may introduce heterogeneity in the BLE‐CRD relationship. Nevertheless, both BLE and CRD showed strong direct associations with SP, consistent with their complementary roles in technology‐related sleep disruption.

### 3.4 Node Predictability

As Table 1 shows, the predictability of node SP was *R* ^2^ = 0.735, indicating that 73.5% of the variance in Sleep Problems could be explained by its neighboring nodes in the network. This high predictability demonstrates that the network structure effectively captures the factors contributing to sleep problems. The mean node predictability across all nodes was *R* ^2^ = 0.65 (range: 0.57–0.74), suggesting that, on average, 65% of the variance in each node could be explained by its network neighbors—indicating a well‐connected and interdependent network structure.

### 3.5 Centrality Analyses

**FIGURE 2:**
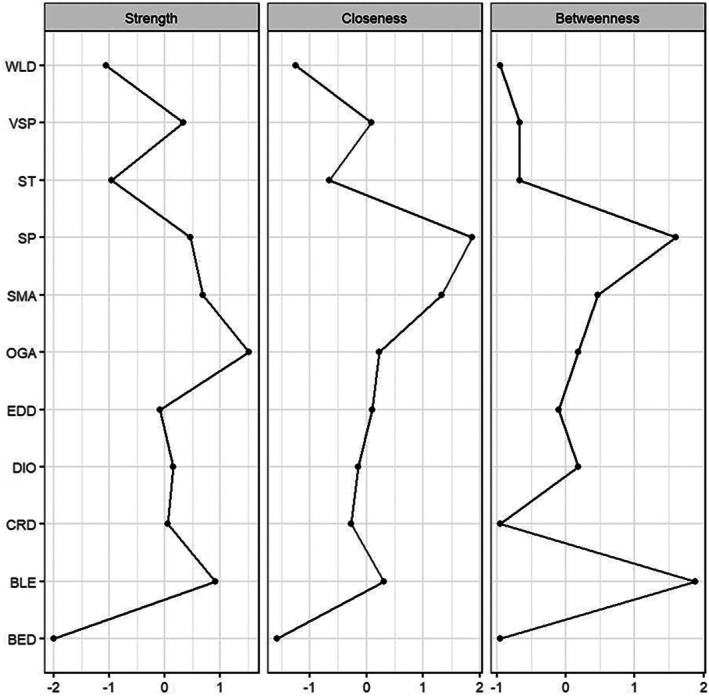
Centrality measures (strength, betweenness, and closeness) for sleep problems network nodes.

Figure 2 and Table 1 display the centrality indices for each node. The node with the highest strength centrality was Online Gaming Addiction (OGA) (*z* = 1.52), followed by Blue Light Exposure (BLE) (*z* = 0.91), indicating that these nodes have the strongest overall connections within the network. The node with the highest betweenness centrality was Blue Light Exposure (BLE) (*z* = 1.88), followed by Sleep Problems (SP) (*z* = 1.60), suggesting that these nodes serve as critical bridges through which other variables in the network are interconnected. The node with the highest closeness centrality was Sleep Problems (SP) (*z* = 1.86), indicating that changes in this node would propagate most rapidly to other variables in the network.

Centrality indices are presented as standardized z‐scores (*x*‐axis), with higher values indicating greater centrality. The *y*‐axis lists all 11 network nodes. Strength centrality reflects the sum of absolute edge weights connected to each node. Betweenness centrality indicates how often a node lies on the shortest path between other nodes. Closeness centrality represents the inverse of average shortest path length to all other nodes. Node abbreviations are defined in Figure 1 legend.

The bootstrap analysis indicated good stability of the network estimates. The correlation stability coefficient (CS‐coefficient) for strength centrality was 0.67, for betweenness centrality was 0.44, and for closeness centrality was 0.52, all exceeding the recommended threshold of 0.25 and indicating that the centrality estimates are sufficiently stable for interpretation [17].

### 3.6 Hypothesis Testing Summary

The results provided support for all three hypotheses. Consistent with H1, Blue Light Exposure demonstrated the strongest direct edge weight with Sleep Problems (*r* = 0.31), supporting its role as the primary proximal factor. Consistent with H2, Screen Time, Bedtime Electronic Device Use, Electronic Device Dependence, Virtual Social Pressure, and Work‐Life Digital Integration showed substantially weaker direct edge weights with SP (*r* range: 0.03–0.12) compared to their zero‐order correlations, indicating that their associations with sleep problems are largely mediated through other network variables. Consistent with H3, Online Gaming Addiction, Digital Information Overload, Social Media Anxiety, and Circadian Rhythm Disturbance showed moderate centrality indices and served as intermediate nodes connecting distal technology factors to sleep problems.

## 4 Discussion

This study employed network analysis to examine the complex relationships among digital technology use factors and sleep problems in a large sample of Chinese adults. The network approach revealed that technology‐related sleep disruption operates through an interconnected system of physiological, psychological, and behavioral factors, with 73.5% of the variance in sleep problems explained by network neighbors. These findings advance theoretical understanding of technology‐induced sleep disruption and provide empirical guidance for intervention development.

### 4.1 Blue Light Exposure as a Proximal Factor

Consistent with our first hypothesis, Blue Light Exposure exhibited the strongest direct edge weight with Sleep Problems and demonstrated high centrality across all three indices. This finding aligns with established chronobiological research demonstrating that evening blue light exposure suppresses melatonin secretion through activation of intrinsically photosensitive retinal ganglion cells. Because melatonin serves as the primary physiological signal for sleep initiation, its suppression delays the onset of sleepiness and prolongs sleep latency, ultimately leading to delayed sleep onset and reduced sleep efficiency [2, 3, 30]. The high betweenness centrality of BLE indicates that it serves as a critical bridge in the network, through which effects of other technology use factors may be channeled.

The strong direct association between Blue Light Exposure and Sleep Problems, even after controlling for all other network variables, suggests that light‐based interventions may be particularly effective for technology‐related sleep disruption. Practical strategies include the use of blue light filtering software, hardware‐based blue light reduction features, and behavioral recommendations to limit screen exposure during the evening hours [31]. The network structure also suggests that reducing blue light exposure may have downstream effects on other network nodes through its bridging role.

### 4.2 Intermediate Factors: Psychological and Behavioral Bridges

The network analysis revealed that Online Gaming Addiction, Digital Information Overload, Social Media Anxiety, and Circadian Rhythm Disturbance function as intermediate factors with direct pathways to Sleep Problems and connections to more distal technology use variables. This finding supports our third hypothesis and provides insight into the mechanisms through which broader patterns of digital behavior translate into sleep disruption.

The significant negative partial correlation between Online Gaming Addiction and Social Media Anxiety (*r* = −0.19) suggests a potential compensatory relationship—individuals with high gaming engagement may use gaming as a coping mechanism to manage or escape from social anxiety [32]. This interpretation is consistent with research showing that immersive gaming activities can provide temporary relief from real‐world stressors and social pressures. However, gaming addiction independently contributes to sleep problems through mechanisms including pre‐sleep cognitive arousal, time displacement, and disrupted sleep schedules [33].

The positive association between Digital Information Overload and Circadian Rhythm Disturbance (*r* = 0.21, *p* < 0.001) highlights the cognitive pathway through which excessive digital information consumption affects sleep. Information overload may maintain cognitive activation during evening hours, interfering with the natural wind‐down process necessary for sleep initiation [34]. This pre‐sleep hyperarousal state characterized by racing thoughts, difficulty disengaging from information streams, and heightened alertness represents a key mechanism through which psychological factors mediate the technology‐sleep relationship.

Social Media Anxiety showed a moderate direct edge weight with Sleep Problems (*r* = 0.20), indicating that anxiety specifically related to social media use contributes to sleep disruption beyond its associations with other network variables. This effect likely operates through psychological mechanisms including rumination about social interactions, anticipatory anxiety about missing updates, and social comparison processes that elevate emotional arousal before sleep [6, 35]. Importantly, this psychological pathway is distinct from the physiological pathway of light exposure—while light affects sleep through melatonin suppression and circadian signaling, social media anxiety affects sleep through emotional and cognitive arousal mechanisms that impair the psychological wind‐down necessary for sleep initiation.

### 4.3 Distal Factors: Indirect Pathways to Sleep Problems

Supporting our second hypothesis, Screen Time, Bedtime Electronic Device Use, Electronic Device Dependence, Virtual Social Pressure, and Work‐Life Digital Integration demonstrated substantially weaker direct edge weights with Sleep Problems compared to their zero‐order correlations. This pattern indicates that these factors influence sleep primarily through indirect pathways mediated by intermediate variables.

The dramatic attenuation of the Screen Time‐Sleep Problems association (from *r* = 0.42 to *r* = 0.03) illustrates a key insight from network analysis: much of what appears to be a direct effect of total screen time on sleep is actually mediated through specific aspects of technology use, particularly Blue Light Exposure and Circadian Rhythm Disturbance. Notably, our Screen Time measure assessed general daily device use across all contexts rather than evening‐specific use, which may explain its weak direct association with sleep problems—the sleep‐disruptive effects of screen time appear to operate primarily through evening‐specific behaviors captured by other variables in the network (BED, BLE). This finding has important implications for intervention design, suggesting that general recommendations to “reduce screen time” may be less effective than targeted interventions addressing specific mediating mechanisms, particularly those involving evening technology use.

Electronic Device Dependence showed moderate direct and indirect effects on sleep problems, consistent with research linking technology addiction to sleep disruption through multiple pathways including time displacement, pre‐sleep arousal, and disrupted routines [36]. Virtual Social Pressure and Work‐Life Digital Integration contribute to sleep problems through their effects on evening technology use patterns, psychological arousal, and boundary management between work and rest periods [37].

### 4.4 Theoretical and Practical Implications

The network approach employed in this study advances theoretical understanding of technology‐induced sleep disruption by revealing the interconnected structure of contributing factors. Rather than conceptualizing technology effects on sleep as a set of independent risk factors, the network model suggests a complex system in which physiological mechanisms (blue light, circadian disruption), psychological factors (anxiety, information overload), and behavioral patterns (gaming, device dependence) mutually influence each other and collectively determine sleep outcomes.

For clinical practice and public health intervention, these findings suggest several priorities. First, interventions targeting Blue Light Exposure through technological solutions (filtering software, device settings) or behavioral recommendations (screen‐free periods before bed) may be particularly effective given its strong direct association with sleep problems and its bridging role in the network. Second, addressing intermediate factors such as Social Media Anxiety and Digital Information Overload through cognitive‐behavioral techniques may help interrupt the psychological pathways through which technology use affects sleep. Third, the finding that general screen time shows minimal direct effects on sleep after controlling for specific mechanisms suggests that interventions should focus on modifying specific aspects of technology use rather than advocating for blanket reductions in technology engagement.

### 4.5 Limitations and Future Directions

Several limitations warrant consideration. First, the cross‐sectional design precludes causal inference about the direction of relationships. While network analysis reveals the conditional independence structure among variables, it cannot distinguish between causal effects, reverse causality, or reciprocal relationships. Longitudinal network analyses employing time‐series data would enable examination of temporal dynamics and more confident causal inference [38].

Second, all measures were self‐reported, which may introduce recall bias and social desirability effects. While we employed validated scales with demonstrated psychometric properties, future research should incorporate objective measures of technology use (e.g., smartphone tracking data, blue light sensors) and sleep (e.g., actigraphy, polysomnography) to strengthen the validity of findings [39].

Third, our sample comprised general population adults recruited through an online platform, which may limit generalizability to clinical populations or individuals without internet access. The exclusion of individuals with diagnosed sleep disorders, while methodologically appropriate for examining technology‐specific effects, means our findings may not generalize to clinical samples. Future research should examine whether the network structure differs in populations with established sleep pathology.

Fourth, the network analysis approach, while providing valuable insights into the structure of relationships among variables, does not capture potential nonlinear effects or threshold phenomena that may characterize technology‐sleep relationships. Future research employing machine learning approaches may complement network analysis by identifying nonlinear patterns and interaction effects.

Fifth, while our analysis focused on technology use as a predictor of sleep problems, the relationship may be bidirectional. Individuals with pre‐existing sleep difficulties may engage in increased digital behavior as a coping mechanism or due to an inability to fall asleep. This reverse causality cannot be ruled out in our cross‐sectional design and warrants investigation through longitudinal studies that can disentangle the temporal sequence of technology use and sleep disturbances.

## 5 Conclusions

This study employed network analysis to examine the complex relationships among digital technology use factors and sleep problems. The findings demonstrate that Blue Light Exposure occupies a central position in the network, showing the strongest direct association with Sleep Problems and serving as a critical bridge connecting other technology factors to sleep outcomes. Intermediate factors including Circadian Rhythm Disturbance, Social Media Anxiety, Online Gaming Addiction, and Digital Information Overload bridge the relationship between distal technology use patterns (Screen Time, Device Dependence, Virtual Social Pressure) and sleep problems.

These findings suggest that technology‐induced sleep disruption is a multidimensional phenomenon requiring comprehensive approaches that address physiological mechanisms (light exposure, circadian regulation), psychological factors (anxiety, cognitive overload), and behavioral patterns (device use habits, boundary management). The identification of Blue Light Exposure as a central intervention target, combined with the recognition of multiple indirect pathways through which technology affects sleep, provides empirical guidance for developing effective, targeted interventions to address sleep problems in the digital age.

## Author Contributions

H.G. and L.X. contributed to the data acquisition and drafted the manuscript. C.T. contributed to the data acquisition. L.X. and C.T. contributed to the study design and revised the manuscript. All authors contributed to the article and approved the submitted version.

## Funding

This research was supported by the Medical and Health Research Projects of Health Commission of Zhejiang Province (2025KY402).

## Ethics Statement

The study has been reviewed and approved by the Ethics Committee of Shaoxing Second Hospital (2024031). The committee verified that all methods used in this study were carried out in line with the 1964 Helsinki declaration and its subsequent revisions or similar ethical standards, as well as the ethical requirements of the institutional research committee.

## Consent

Informed consent has been obtained from all subjects involved in this study.

## Conflicts of Interest

The authors declare no conflicts of interest.

## Data Availability

The datasets used and/or analyzed during the current study are available from the corresponding author on reasonable request.
